# Death of Dr. Hamilton

**Published:** 1899-01

**Authors:** 


### Editorial Department.

F. E. DANIEL, M. D., Editor.
S. E. HUDSON, M. D., Managing Editor.
A. J. SMITH. M. D., Galveston, Associate Editor.

Published Monthly at Austin, Texas, by Drs. Daniel and Hudson. Subscription price $1.00 a year in advance.

Eastern Representative: John Guy Monihan, St. Paul Building, 220 Broadway New York City.

Official organ of the West Texas Medical Association, the Houston District Medical Association, the Austin District Medical Society, the Brazos Valley Medical Association, the Galveston County Medical Society, and several others.

Death of Dr. Hamilton.—Dr. Jno. B. Hamilton, so well known to the profession and the public died at Elgin, Illinois, December 24th, ult., of peritonitis.

Dr. Hamilton filled a larger measure of usefulness in his profession than perhaps any man in it. His loss is a cause for deep and profound grief, and there are few who read this who will not agree with us that it is a public calamity. He was in the prime and vigor of mature manhood, and being a man of fine physical health, and of the most rare and brilliant attainments, he bid fair to fill out the allotted term of three score years and ten, leaving the world wiser and better for his works. He was doubtless the foremost sanitarian in America, and one of the most learned men in the history and literature of medicine that have graced the century. His address on the "History of Medicine" is monumental. It will be recalled by all readers of the *Journal of the American Medical Association*, which periodical he edited and managed with such signal ability and success. It was a positive luxury to read his editorials.

Dr. Hamilton, in addition to the great labors of the *Journal* of the Association, filled the Chair of Surgery in Rush Medical College, and was Superintendent of the Illinois State Insane Asylum. He had also, recently, in recognition of his scholarly attainments, been elected President of the Illinois State Library Association.

Dr. Hamilton entered the United States army as surgeon in 1874, and in a few years had risen to be the Surgeon-General of the Marine Hospital Service, being appointed by the president upon the death of Dr. Woodward. This position he held twelve years, and resigned to take the position of surgeon in that service with the understanding that he should be stationed at Chicago, where he had accepted a professorship in Rusk Medical College, and hold that position two presidential terms. It was with this understanding, Dr. Hamilton informed the writer, that he recommended the present incumbent, Dr. Walter Wyman, for his successor. He resigned from the service when Dr. Wyman ordered him to San Francisco, the readers of the *Journal* will recollect.

As illustrating the esteem in which Dr. Hamilton was held by the United States government, when the cholera threatened us in 1892, and it became necessary to establish a special quarantine against it, the president, instead of entrusting the task to the Surgeon-General of the Marine Hospital Service, summoned Dr. Hamilton—the ex-Surgeon-General—from Chicago, and put the work in his hands. He kept the cholera out.

Dr. Hamilton was earnestly engaged in the effort to secure the creation by Congress of a Department of Public Health, and had he lived no doubt his efforts would have been successful. The JOURNAL had predicted it, and hoped to see him fill the position of the first Secretary of Public Health—a cabinet officer.

Dr. Hamilton was only 51 years of age.

